# Three-dimensional images contribute to the diagnosis of mucous retention cyst in maxillary sinus

**DOI:** 10.4317/medoral.18141

**Published:** 2012-12-10

**Authors:** Cleomar Donizeth-Rodrigues, Márcia Fonseca-Da Silveira, Ana H. Gonçalves-De Alencar, Maria A. Garcia-Santos-Silva, Elismauro Francisco-De-Mendonça, Carlos Estrela

**Affiliations:** 1 DDS, MSc, PhD Professor, Radiology, Brazilian Dentistry Association, Brasília, Brazil; 2 DDS, MSc, PhD Chairman and Professor, Diagnosis, University of Pernambuco, Recife, Brazil; 3 DDS, MSc, PhD Professor, Endodontics, Federal University of Goiás, Goiânia, Brazil; 4 DDS, MSc, PhD Professor, Radiology, Federal University of Goiás, Goiânia, Brazil; 5 DDS, MSc, PhD Chairman and Professor, Radiology, Federal University of Goiás, Goiânia, Brazil; 6 DDS, MSc, PhD Chairman and Professor, Endodontics, Federal University of Goiás, Goiânia, Brazil

## Abstract

Objective: To evaluate the detection of mucous retention cyst of maxillary sinus (MRCMS) using panoramic radiography and cone beam computed tomography (CBCT). Study Design: A digital database with 6,000 panoramic radiographs was reviewed for MRCMS. Suggestive images of MRCMS were detected on 185 radiographs, and patients were located and invited to return for follow-up. Thirty patients returned, and control panoramic radiographs were obtained 6 to 46 months after the initial radiograph. When MRCMS was found on control radiographs, CBCT scans were obtained. Cysts were measured and compared on radiographs and scans. The Wilcoxon, Spearman and Kolmorogov-Smirnov tests were used for statistical analysis. The level of significance was set at 5%. Results: There were statistically significant differences between the two methods (p<0.05): 23 MRCMS detected on panoramic radiographs were confirmed by CBCT, but 5 MRCMS detected on CBCT images had not been identified by panoramic radiography. Eight MRCMS detected on control radiographs were not confirmed by CBCT. MRCMS size differences from initial to control panoramic radiographs and CBCT scans were not statistically significant (p= 0.617 and p= 0.626). The correlation between time and MRCMS size differences was not significant (r = -0.16, p = 0.381). Conclusion: CBCT scanning detect MRCMS more accurately than panoramic radiography.

** Key words:**Mucous cyst, maxillary sinus, panoramic radiograph, cone beam computed tomography.

## Introduction

Mucous retention cysts of the maxillary sinus (MRCMS) are an asymptomatic lesion incidentally found during the examination of images. On radiographs, they are radiopaque, dome-shaped structures with a distinctly rounded edge. They are slow growing lesions, but mucosal and cortical integrity is preserved (1). Their etiology is unclear (2,3): They may be associated with allergic and inflammatory processes of the nasal sinus mucosa (1,4-6), trauma (7), periapical and periodontal infections (2,4,8,9), and relative humidity and room temperature (3,4,10). However, no significant correlation has been found between relative humidity, mean temperature and month of diagnosis of MRCMS (11). Because its rate of spontaneous regression and disappearance is 16% to 41% (2,4,12), it should be followed up clinically and radiographically, but even when it increases considerably, no specific treatment should be used, except for relief of symptoms, if necessary (12).

Imaging examinations provide opportunities for dentists to detect changes in the maxillary sinus. A Waters’ view is considered ideal for the evaluation of the maxillary sinuses, but the most inferior and posterior aspects may not be unclear because of the overlap of the alveolar process and the posterior teeth (13). Panoramic radiography has been used as a routine screening tool for the evaluation of the maxillomandibular complex. Although not suitable for evaluating maxillary sinuses along all their extension (14) because of its limitations, panoramic radiography is still used because of its low cost, availability and ease of interpretation (13).

Computed tomography (CT) is a valuable diagnostic method to examine the paranasal sinuses (15). Martínez-González et al. (16) compared panoramic radiography and CT to evaluate 84 maxillary sinuses and found that panoramic radiography had limitations in the diagnosis of changes in maxillary sinus, whereas CT seemed to be a better imaging tool. Despite the advantages of CT, it is no longer used in routine dental care because of its high radiation doses and cost (16-18).

Cone beam computed tomography (CBCT) (17,19), a more recent technological development, reproduces mineralized maxillofacial tissues as three-dimensional images with minimal distortion and radiation doses that are significantly lower than that of CT (17-19). CBCT may become an important tool to diagnose changes and plan the treatment of maxillary sinus alterations (20).

Few studies have compared the use of panoramic radiography and CBCT to detect changes in the maxillary sinuses. This study evaluated the detection of mucous retention cysts of the maxillary sinus using panoramic radiography and cone beam computed tomography.

## Study Design

Six thousand panoramic radiographs obtained between October 2006 and June 2010 for purposes of dental treatment were selected from the digital database of a private radiology clinic (Revelação Imagens Orais, Brasília, Brazil). The inclusion criteria were: properly acquired and processed radiographic images; and patient age 12 years or older. We selected 185 radiographs with suggestive images of MRCMS, and patients were located and invited to return for follow-up. Thirty two returned and agreed to participate in the study. We excluded two patients: one had undergone maxillary sinus surgery and the other was pregnant.

This study was approved by the Ethics in Research Committee of Federal University of Goiás, Brazil, under protocol 169/2009. Participants signed an informed consent term.

Control panoramic radiographs were obtained for 30 patients, and when a suggestive image of MRCMS was found, the patient was asked to undergo CBCT for a better evaluation of the maxillary sinus.

**Figure 1:**
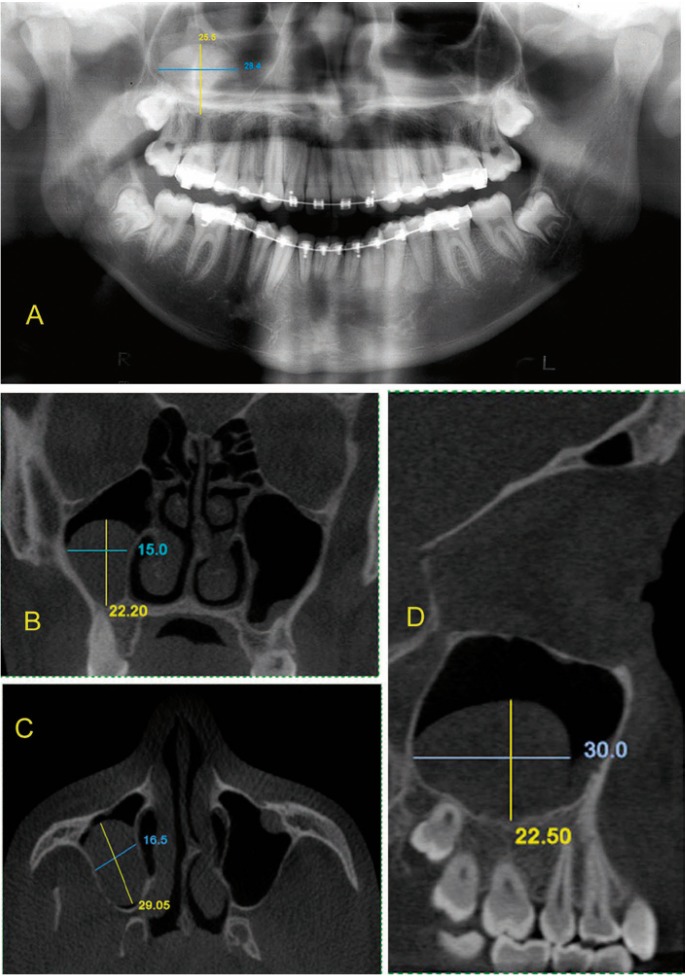
Measurement of MRCMS on panoramic radiograph A) and on coronal B), axial C) and sagittal D) CBCT reconstructions of same patient.

Initial and control panoramic radiographs were obtained using an Orthoralix 9200 AEC panoramic system (Gendex® Dental Systems, Des Plaines, IL) using 0.5 mm focal spot and Kodak film (T-MAT, 15X30, Manaus, Brazil). Images were stored in digital JPEG format at 150 dpi after scanning using a Scan Jet 4C HP® with a transparency unit. Two specialists in Dental Radiology and Imaging with over 10 years’ clinical experience were previously calibrated. They analyzed the images to detect MRCMS and, when there were differences between their evaluations, a consensus was reached by discussing the image with a third specialist in radiology. The criterion for MRCMS detection using panoramic radiographs was the visualization of a dome-shaped radiopaque image on the floor or other walls of the maxillary sinus. Superoinferior and lateromedial measurements of MRCMS were made on initial and control panoramic radiographs using the software Radiocef Studio 2 (Radiomemory®, Belo Horizonte, Brazil) according to the longest dimension (Fig. 1).

The CBCT images were obtained using an i-CAT scanner (Imaging Sciences® International, Hatfield, PA, USA), at 120 kVp, 18.45 mAs, and exposure of 20 seconds. Field of view (FOV) was 13 cm (from the crowns of upper teeth to the middle third of frontal bone), voxel size was 0.3 x 0.3 x 0.3 mm, and gray scale was 12 bits.

The images in DICOM format were processed, interpreted and measured using Xoran Cat 3.1.62 (Xoran® Technologies, Ann Arbor, MI). MRCMS detection criterion using CBCT was the view of a dome-shaped opaque structure on the floor or other walls of the maxillary sinus. Measurements of MRCMS were made in the sagittal, axial and coronal reconstructions considering the greatest dimension (Fig. 1).

Radiographs and CBCT scans were evaluated using an Intel® CoreTM 2 Duo-6300 computer, 2.00 GHz, 2.93 GB RAM (Intel Corporation, Santa Clara, CA), NVIDIA GeForce 6200 Turbo Cache videocard (NVIDIA ® Corporation, Santa Clara, CA) and a 19-inch EIZO monitor, FlexScan S2000, 1600x1200 pixels (EIZO NANAO® Corporation, Hakusan, Japan) in an adequate room. The differences between MRCMS dimensions on initial and control panoramic radiographs and between control panoramic radiograph and CBCT scan was obtained by calculating the difference between the greatest dimensions.

To analyze the frequency of MRCMS according to diagnostic method, the Kolmorogov-Smirnov test was used (p<0.05). The Wilcoxon test was used to evaluate the differences between initial and control panoramic radiographs, and between control panoramic radiographs and CBCT scans. The correlation between control time and MRCMS size was analyzed using the Spearman test.

Patients who had other sinus pathologies were referred to specialized care and those with MRCMS have been regularly followed up. 

## Results

The mean age of the 30 patients who underwent radiographic control was 37.5 years, and 17 were men ( Table 1). The time interval between initial and control panoramic radiographs was 6 to 46 months.

**Table 1:**
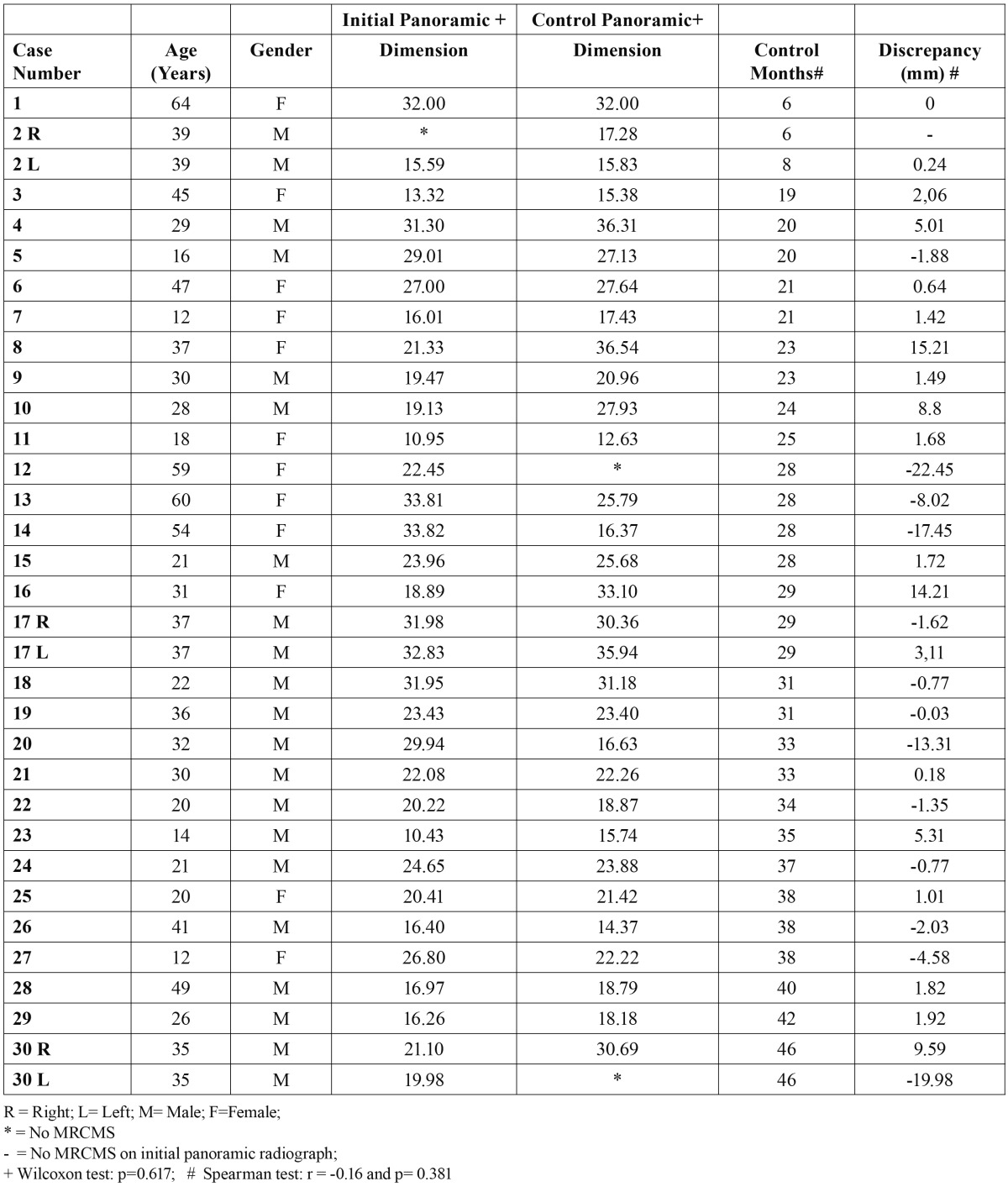
Dimension (mm) and control time (months) of MRCMS detected on initial (n=32) and control (n=31) panoramic radiographs.

Thirty-two MRCMS were detected on the initial panoramic radiographs; 28 were unilateral, and 2, bilateral. On control panoramic radiographs, 31 MRCMS were found (27 unilateral and 2 bilateral): 2 initial MRCMS disappeared, and a new one was detected.

Differences of MRCMS size on initial and control panoramic radiographs ranged from -22.45 (reduction or disappearance of MRCMS) to +15.21 mm (increase of MRCMS size). No differences were statistically significant (Wilcoxon test; p= 0.617).

On the control panoramic radiographs, 46.87% (n=15) of the MRCMS were larger, 25% (n=8) were smaller, 21.87% (n=7) re-mained unchanged or had less than 1-mm changes, and 6.25% (n=2) disappeared ( Table 1).

The correlation between elapsed time from initial to control panoramic radiograph and MRCMS differences were analyzed using the Spearman test, and the results were not statistically significant (r = -0.16, p= 0.381).

**Figure 2:**
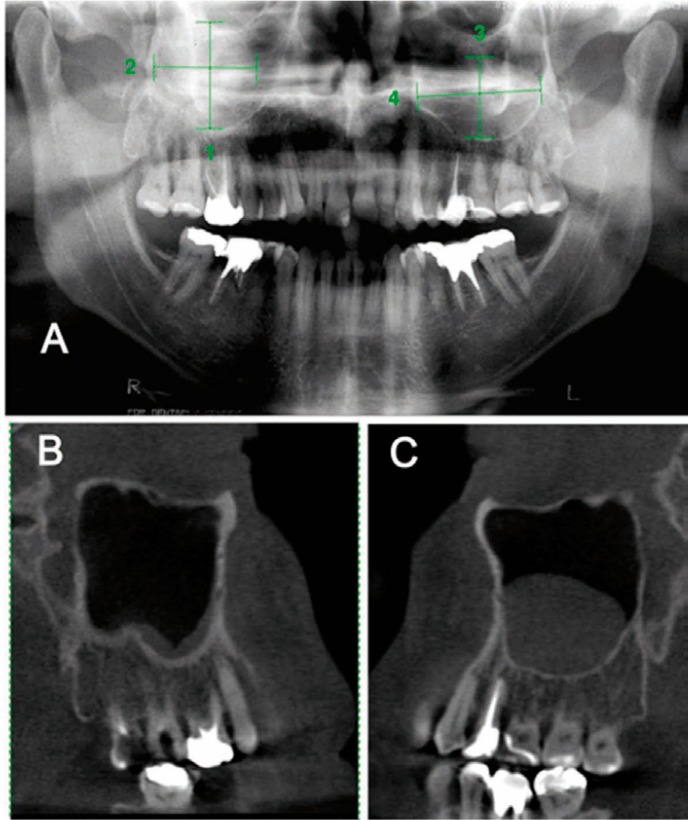
Control panoramic radiograph of bilateral MRCMS A), sagittal CBCT reconstruction of same patient, with no MRCMS in right side B) and MRCMS in left side C).

**Figure 3:**
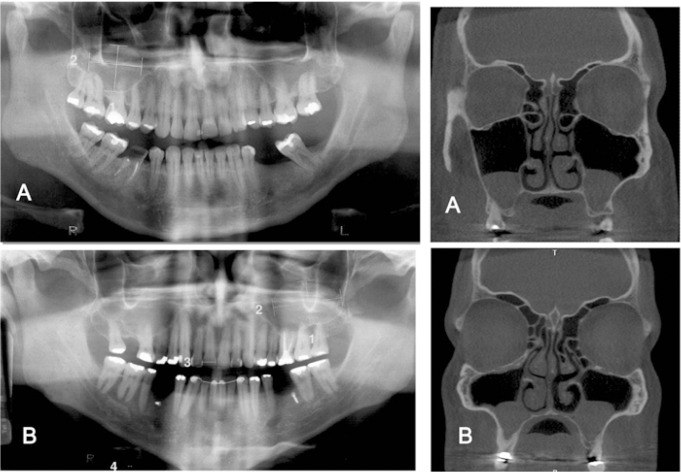
A) Control panoramic radiograph with MRCMS in right side and coronal CBCT reconstruction confirming MRCMS in right side and showing another MRCMS in left side. B) Control panoramic radiograph with MRCMS in left side and coronal CBCT reconstruction of same patient with bilateral MRCMS.

Of the 31 MRCMS detected on control panoramic radiographs, 23 were confirmed on CBCT images, and 8 were false positive (Fig. 2). CBCT images identified 5 MRCMS not detected on the control panoramic radiographs (Fig. 3). The frequency of MRCMS detected by control panoramic radiography and CBCT was assessed using the Kolmogorov-Smirnov test, and the differences were statistically significant (p<0.05).

Of the 23 MRCMS detected by panoramic radiography and confirmed by CBCT, 12 (52.17%) were larger on CBCT scans, 5 (21.73%) were smaller, and 6 (26.08%) had the same size, but these findings were not statistically significant (Wilcoxon test; p = 0.626) ( Table 2).

**Table 2:**
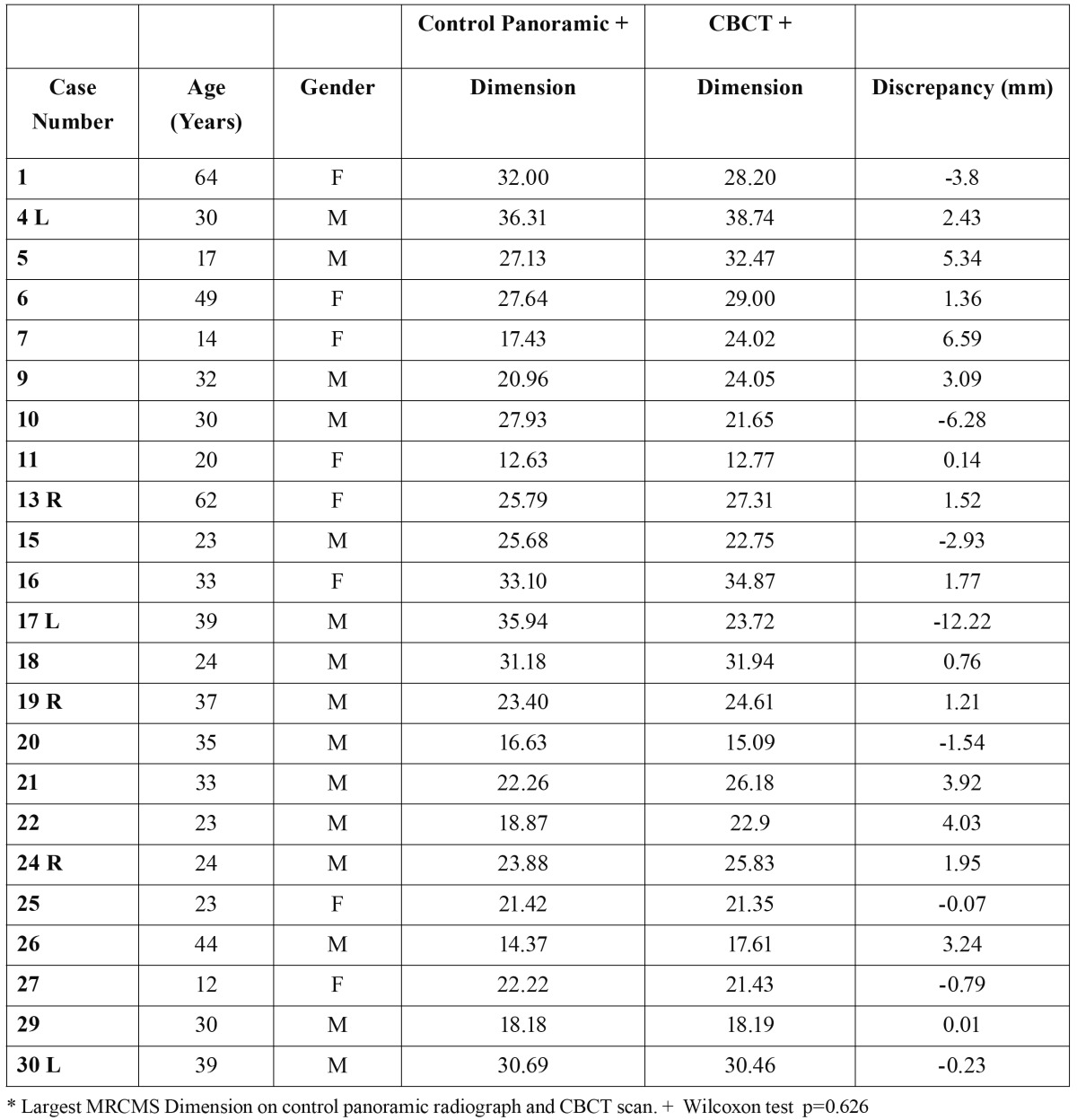
Dimension (mm) of MRCMS detected on control panoramic radiograph and on CBCT scan (n = 23).radiographs.

## Discussion

Imaging detection of MRCMS may help to define its characteristics and behavior, as well as to establish a therapeutic protocol. MRCMS does not affect the integrity of maxillary sinus walls (1) and is usually asymptomatic (7,12,15,21); in most cases, it resolves spontaneously and requires no treatment (12). Clinical and radiographic examinations are essential to define alternative treatments and to rule out other pathologies, such as mucocele, polyps and sinusitis (4,15).

In this study 32 MRCMS were detected on initial panoramic radiographs and 31 on control panoramic radiographs; 2 MRCMS seen on initial panoramic radiographs disappeared, and a new one was diagnosed. There were no statistically significant differ-ences between MRCMS size on initial and control panoramic radiographs, and there was no correlation between MRCMS size and time between examinations.

Wang et al. (12) reported that when MRCMS shows no significant changes in four years, it will probably have the same size at a later date. If a significant increase is observed, it can be expected to be larger at a second control examination. As the rate of spontaneous regression and disappearance of MRCMS varies between 16% and 41% (2,4,12), only clinical and radiographic follow-up is recommended, and no specific treatment should be prescribed even when considerable increase is noticed, except to relieve possible symptoms (12).

The results of this study showed significant differences in the identification of MRCMS using CBCT and panoramic radiography. Twenty-three MRCMS detected using panoramic radiography were confirmed by CBCT; however, 5 MRCMS detected on CBCT scans had not been identified on the panoramic radiographs. These results may be assigned to the limitations of panoramic radiographs, which do not show the entire length of the maxillary sinus. The roof of the maxillary sinus and minor changes located outside the imaging window and in superolateral regions or in the center of the maxillary sinus cannot be viewed (13,22,23).

Panoramic radiographs in this study had images suggestive of 8 MRCMS that were not confirmed on CBCT scans. Despite the benefits, panoramic radiography has limitations, such as image overlay, which may lead to false positive results. Lower nasal concha and nasal cavities extend and protrude over the maxillary sinus when the patient is positioned too far from the X-ray machine or with the head raised, which produces images that suggest changes in the maxillary sinuses (24). A previous study compared CT with panoramic radiography and concluded that CT remains the most effective test for the diagnosis of inflammatory changes of the maxillary sinuses (16).

The development of CBCT equipment has resulted in better image quality for diagnoses, exposure to lower radiation doses, easier operation and lower cost than CT (17-19,25,26). CBCT may be a useful tool for diagnosis and treatment planning of maxillary sinus diseases (20). This study compared panoramic radiographs with CBCT images and found that, of the 23 MRCMS detected by panoramic radiography and confirmed by CBCT, 12 (52.17%) were larger, 5 (21.73%) were smaller, and 6 (26.08%) remained the same. These results were supported by the fact that the greatest dimension of many MRCMS was in the posteroanterior direction on CBCT scans, a measurement that could not be made on panoramic radiographs because conventional radiographic images have only two dimensions. CBCT images provided readings by mapping and acquisition of valuable information by viewing at different levels.

In conclusion, in the comparison with panoramic radiographs, CBCT, which has led to significant advances in Dentistry diagnosis and research, detected MRCMS at a greater precision than radiography.
